# Supplementary material for: Selective transport of water molecules through interlayer spaces in graphite
Source: Nat Commun. 2022 Jan 25;13:498. doi: 10.1038/s41467-022-28162-6 (PMC8789919; doi:10.1038/s41467-022-28162-6)
Supplement: Supplementary file 1 — Supplementary Information [file 41467_2022_28162_MOESM1_ESM.pdf]

Supplementary Information

Selective transport of water molecules through interlayer spaces in graphite

L. Saini *et al.*

Supplementary Note 1. Sample Preparation, Wetting and cleaning procedures:

For the intercalation, we have used a block of highly oriented pyrolytic graphite (HOPG) (size 12 mm × 12 mm × 2 mm) commercially obtained from Materials Quartz, Inc, USA. The as-obtained HOPG was then carefully cut into smaller pieces with sizes 6 mm × 6 mm × 0.25 mm with the help of a scalpel blade. This was then transferred onto an acrylic sheet that has a pre-prepared square hole of size 2 mm × 2 mm. This acrylic sheet serves as the mechanical support for the transferred HOPG, and the HOPG is tightly sealed with the help of Stycast epoxy glue (inset of Fig. 1b). The samples were left to dry for a minimum of 12 hours, which is the typical cure time for the epoxy setting. After this, we accurately measured the actual transport area of the graphite with the help of an optical microscope (Nikon Eclipse E200). We also tried several other polycrystalline graphite samples; however, those samples were found to be very leaky and exhibit very high ion currents with very small applied voltages, making them unsuitable for the intercalation study.

We wetted the surface of the sample at the beginning of each measurement. For this, we immersed the sample in 2-Propanol (IPA) for about 30 minutes. Prior to each set of measurements, the electrochemical cell was washed sequentially with IPA (100), IPA + DI water (50:50) and DI water (100) for proper wetting of the sample and removal of any residual salts. Ion transport measurements that utilize different salt concentrations the measurements were always taken from low concentrations to high. Between the measurements with different salt solutions, the cell was rinsed with DI water multiple times until the current observed through graphite was equal to the initially measured current for DI water. This data also helped us to monitor the stability of the devices. We found that in the successfully intercalated samples, more than 90% of the devices were water stable with no indication of any degradation in the transport characteristics. These sub-nm channels are very sensitive to molecules' adsorption, which leads to blockage of channels in the dry state, so it is essential to keep the samples in water all the time.

Supplementary Note 2. Ion-transport studies:

An electrochemical cell was custom-made with PEEK (polyether ether ketone) material for the ion transport studies. The cell has two reservoirs with a capacity of 5 ml each. The sample is placed in the middle of two reservoirs, held firmly on two O-rings on both sides of the sample (Supplementary Figure 1). The cell was thoroughly cleaned with acetone, DI water, and IPA in an ultra-sonicator bath for 30 minutes each, before mounting graphite for intercalation. This cleaning procedure is very important to avoid the formation of any air bubbles inside the cell. After thoroughly wetting the sample surface, salt solutions were filled in two reservoirs such that the graphite surface was fully immersed in the solution. The acrylic support and epoxy glue ensured that graphite is the only path for the ions or water molecules. We measured the leakage current of our setup using a blank acrylic sheet. The detected leakage current is

~10^{-12} A for a maximum applied voltage of 200 mV. This current is several orders of magnitude smaller than the recorded values of current through our intercalated graphite samples.

To further check the possibility of any leakage at the interface of an acrylic base and epoxy glue, a voltage similar to that used for intercalation (10 V) was applied to an acrylic piece pasted on a 2 mm × 2 mm hole. The measured current through this sample was still ~10^{-12} A, which is also the lower limit of our measurement setup. This means that the epoxy interface is stable even after applying a high voltage without any generation of defects.

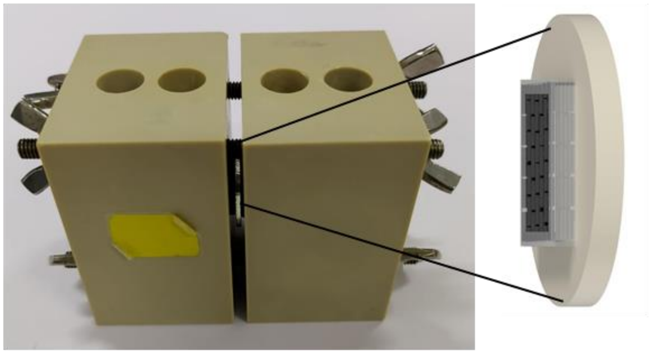

Supplementary Figure 1: Electrochemical cell. The optical image of the custom-made electrochemical cell along with an enlarged view of the graphite sample on an acrylic support.

To estimate the salt conductance, we performed *I-V* measurements and the slope of the curve is taken as the measure of conductance. For this, a voltage is swept in the range of 200 mV, in steps of 25 mV and the resultant current is recorded. At low salt concentrations, we observed a small hysteresis in the *I-V* loop measurements. We could remove this hysteresis by increasing the applied voltage duration from the typical 5 s to 180 s, so that the accumulated charge is completely discharged. The small current through unintercalated graphite indicates high quality of our graphite sample and also the efficacy of epoxy sealing. We note that a similar leakage current was reported in the case of ion transport through blocked BN nanotube assembly[1]. Just after this measurement, we proceeded with the intercalation of 2 M KCl. For this, we have filled both reservoirs with 2 M KCl, and applied a maximum voltage of 10 V for a duration of maximum 3 hours for thick samples. For very thin samples (~30 µm), the optimum time was found to be hundreds of seconds for an applied voltage of 10 V. After the completion of intercalation, the assembly was thoroughly cleaned, which removed any residual salts present at the surface.

The success of the intercalation was inferred from the (i) enhanced ionic current of 1000 times to that of unintercalated samples, (ii) A new peak in the XRD pattern at lower angles, (iii) presence of K and Cl in the SEM-EDAX mapping, (iv) presence of K and Cl in the XPS spectra of intercalated samples (v)

increased water flux. Graphite intercalation was also done with other reported methods[2] and compared against the XRD patterns obtained for our method (Supplementary Figure 2), including H_2SO_4 intercalation of graphite using the secondary intervening method as described in[3]. The sample intercalated with H_2SO_4 did not significantly change the XRD peak intensities though the sample was visibly expanded. This suggests that the success of intercalation is tough to be judged with the XRD technique alone. We also used the electrochemical intercalation method where the graphite is made as one of the electrodes with 2 M KCl as the electrolyte and platinum as a counter electrode. We observed that the graphite electrode expanded very quickly (hundreds of seconds) in this experiment.

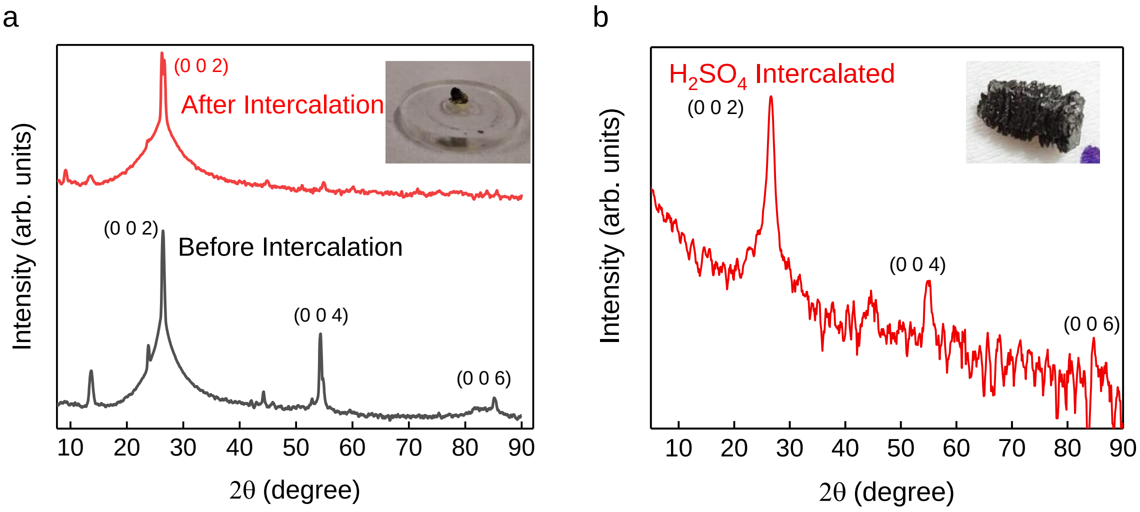

Supplementary Figure 2: **The effect of interlayer distance upon intercalation.** (a) X-ray diffraction (XRD) plot of graphite before and after the KCl intercalation. A new peak at $2\theta = 9.10°$ emerges after the intercalation, suggesting successful intercalation of KCl. Inset shows a graphite device that has been intercalated as indicated from the visible expansion. In addition, several intense peaks at higher angles almost vanished after the intercalation process. (b) XRD pattern of H_2SO_4 intercalated graphite. The inset shows the visibly expanded sample as a result of intercalation.

We have performed contact angle measurements to clarify the surface modifications and the water in-take mechanism (Supplementary Figure 3). The applied voltage helps the entry/exit of the graphite interlayer space become hydrophilic, which helps the water enter the interlayer space via capillary action. The nearly hydrophobic bulk channel induces slip-enhanced water flow.

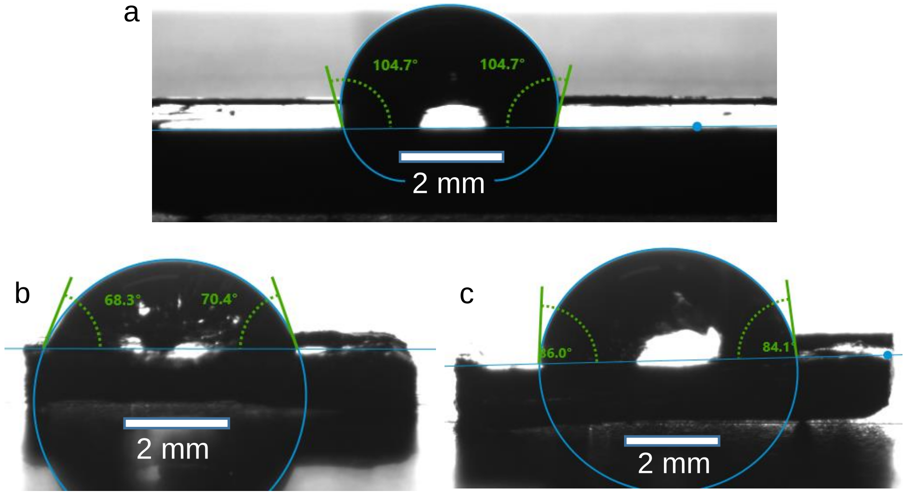

Supplementary Figure 3: Contact angle measurements. (a) The graphite surface is hydrophobic before the intercalation. (b) After the intercalation, the graphite surface becomes hydrophilic. (c) After removing a few top layers of intercalated graphite, the surface is less hydrophobic than the pristine sample.

The repeatability of the results was checked by intercalating approximately 14 samples of similar areas. A histogram showing the variation in water conductance of different samples is shown in Supplementary Figure 4. The water conductance lies in the range of 0.2-2.4 × 10^{-6} S.

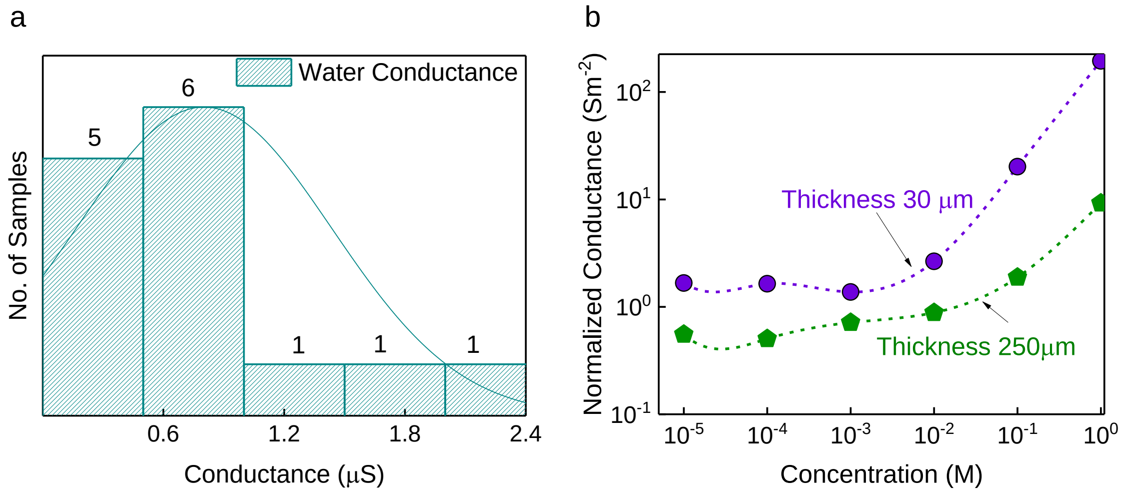

Supplementary Figure 4: Reproducibility of the samples. (a) Histogram showing the water conductance of 14 samples of similar geometrical dimensions after the intercalation. Above each rectangular bar, the sample number is indicated. (b) NaCl conductance (normalized for area) variation with thickness.

Supplementary Note 3. Water evaporation and weight-loss studies:

After the ion-transport studies, the same sample is used for measuring the water evaporation rate. For this, we cleaned the sample with 2-Propanol to remove any salt residues on the surface. After that, the sample is placed inside a liquid cell (Supplementary Figure 5). This cell is made up of PEEK (Polyether ether ketone) and the sample is placed in between two O-rings, which keeps the sample leak-tight. The water evaporation assembly is placed inside a high precision micro balance (Mettler Toledo XSR105) of resolution, 10 µg.

A LabVIEW program connected to the precision balance is used to record weight loss for more than 12 hours, with a 1-minute gap between the readings. For controlling the humidity level, silica gel was heated for 10 minutes under an IR lamp and then kept inside the precision balance, well before the measurements. The same procedure was used to measure the weight loss through all the samples, including ICG, 2 mm × 2 mm hole (reference), and unintercalated graphite sample. In majority of the cases, the weight loss rate through ICG was found to be higher than the reference sample. In very few samples, a smaller weight loss than the reference was also observed within 4 to 8 hours when the water was accidently not in contact with the surface of the sample. For unintercalated graphite, the weight loss was significantly lower than the reference.

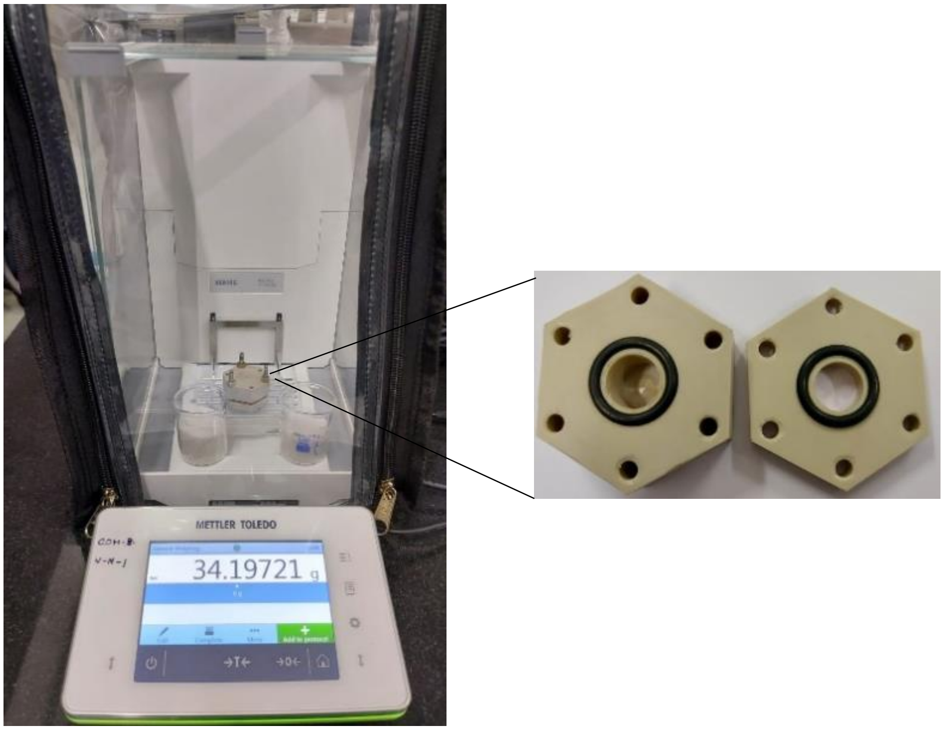

Supplementary Figure 5: Assembly used for the measurement of water evaporation rate. The evaporation rate is estimated from the recorded weight loss at different intervals of time, measured using

a precission weighing balance (left image). The liquid cell that is used for the measurement is shown in the right image.

For ensuring the accuracy of our gravimetric setup, we prepared a reference sample with 2 mm × 2 mm hole drilled through a 2 mm thick acrylic sheet. We measured the water evaporation rate through the open aperture using the gravimetry setup discussed above. For our aperture, the Knudsen number is small and we can safely assume that the water evaporation can be described by diffusion of water molecules through air. The molecular flow is given by[4]

$$F = \frac{1}{3} < v > l \frac{dn}{dx} \qquad \text{Supplementary Equation (1)}$$

Where $< v >$ is average velocity of water molecules in air, taken to be 590 m s^{-1}; $l \approx 60$ nm is the mean free path and dn/dx is the concentration gradient. When leaving the container, water molecules diffuse through air for a distance equal to the thickness of our reference sample (2 mm). Thus, we can write dn/dx = Δn/t. Where Δn is the difference in water concentrations at large distances from the aperture and t is the thickness of our reference sample. Further, Δn can be estimated as $\Delta P/k_B T$; where ΔP is difference in partial pressure, k_B is Boltzmann constant and T is temperature. For solving the diffusion problem exactly, we need to choose between the limit of a steady state of thin film or un-steady state of thick slab[5]. A simple criteria to choose between the two is given in[5], according to which if the ratio

$$\frac{(Length)^2}{(diffusion\ coefficient)(time)}$$

is much less than unity, we can assume that the system is in a steady-state. The measurement time of our evaporation experiment is typically longer than 24 hours, so we can consider this as a steady-state diffusion process, and hence it boils down to the diffusion problem for the case of thin orifices[5]

$$\frac{dn}{dx} = \frac{4}{\pi}\left(\frac{\Delta n}{t}\right) \qquad \text{Supplementary Equation (2)}$$

The resulting weight loss is

$$Q = F M_{H_2O} A \qquad \text{Supplementary Equation (3)}$$

where M_{H_2O} is the molar mass of water and A is the area of the open aperture. Using ΔP as 23 mbar, from the above equation, we get $Q \approx 5.02 \times 10^{-7}$ g s^{-1}, which is in good agreement with the experimentally measured value through the open aperture, indicating the accuracy of our experimental setup.

Supplementary Note 4. Forward osmosis experiment for the estimation of water flux:

Forward osmosis uses osmotic pressure gradient to draw water molecules across a semi-permeable membrane. In this process, draw solution drives water molecules across the sample from feed solution.

Our study used equal amounts of 2 M sucrose and deionized water as draw solution and feed solution, respectively, separated by a sample with an effective area of 3.4 mm^2 and thickness of 0.25 mm. The osmotic pressure (π) is calculated from the van't Hoff equation

$$\pi = \phi iRTM \qquad \text{Supplementary Equation (4)}$$

where M, R and T are molar concentration (mol L^{-1}), universal gas constant and temperature, respectively. ϕ is osmotic coefficient ($\phi_{sucrose}$ = 1.02), and i is the number of ions in which the solvent dissociates ($i_{sucrose}$ = 1). For our setup, these values provide an osmotic potential gradient of ~50 bar. The actual values of pressure might be slightly different due to capillary pressures. We have observed a 100 µL increase in volume after 24 hours, which translates into a water permeation rate of 1.22 L m^{-2} h^{-1}, a value close to that estimated from water evaporation experiments. We have utilized high-precision micro-pipettes to measure small volumes with reasonable accuracy. We also observed changes in water levels from the captured images and method followed in[6]; however, was difficult to estimate small changes in volume accurately.

Supplementary Note 5. Comparison with classical flow equations:

If we assume that water behaves as a classical liquid inside the interlayer spacing, then we can apply the Hagen-Poiseuille equation to estimate the flow rate as

$$Q \approx \frac{h^3\rho}{12\eta}\left(\frac{\Delta P}{L}\right)w \qquad \text{Supplementary Equation (5)}$$

where ρ and η are the density and the viscosity of water, taken as 998 kg m^{-3} and 1 mPa s respectively and L is the thickness of the sample, which is estimated to be 0.25 mm. w, is the width, which is taken as ~2 mm. The areal density of the channels is estimated from the relation $A/(w \times h)$. With vapor pressure as 23 mbar, the estimated flux is ~4 × 10^{-7} L m^{-2} h^{-1}, which is seven orders of magnitude lower than what is observed experimentally. But, if we consider the interaction between water and graphite edges, the effective pressure is assumed to be of capillary in origin, and hence can be taken as 1 bar, just to get a rough idea. The water flux estimated is ~14 µL m^{-2} h^{-1}, four orders of magnitude smaller than the measured value. So, in agreement with the previous reports, a slip correction term must be included in the Hagen-Poiseuille equation due to the hydrophobic nature of graphite walls, which is discussed in the main text.

Supplementary Note 6. Confirmation of KCl intercalation with SEM-EDAX and XPS:

EDAX-SEM analysis of the samples was also carried out, both before and after the intercalation process (Supplementary Figure 6). For this, the samples were sliced from the middle and the cross-section was analyzed. Only carbon was detected in the sample before the intercalation, while after the intercalation,

potassium and chlorine were also detected. A complete mapping of K and Cl shows that it is uniformly distributed across the layers.

We also performed X-ray photoelectron spectroscopy (XPS) (Make, Thermo scientific and Model, ESCALABE 250Xi and Al source) analysis of the samples before and after the intercalation of graphite (Supplementary Figure 7). The presence of K and Cl inside the layers is clearly evident in the intercalated graphite samples and are uniformly distributed across the depth (Supplementary Figure 8).

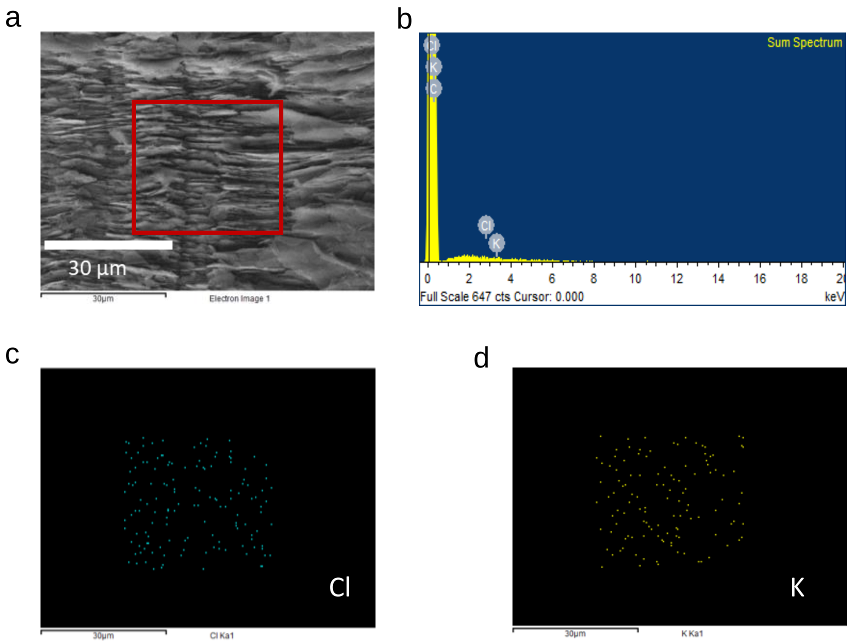

Supplementary Figure 6: SEM-EDAX characterization of samples after intercalation. (a) The cross-section of intercalated graphite. (b) EDAX peaks show the elements present in the area enclosed by the red box in (a). Elemental mapping indicates the presence of chlorine (c) and potassium (d) in the sample.

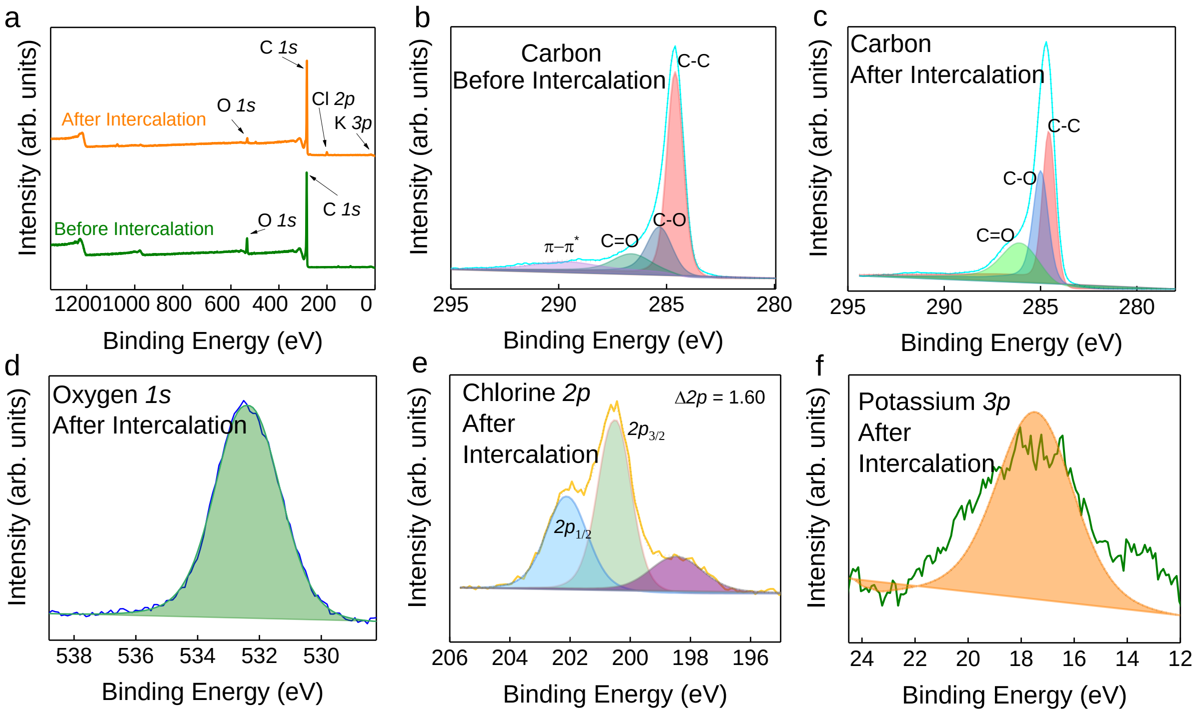

Supplementary Figure 7: X-ray photoelectron spectroscopy (XPS) results. (a) XPS survey of graphite sample before and after the intercalation of KCl. The presence of chlorine and potassium is evident in the intercalated samples, while this was absent in the unintercalated samples. The carbon peaks look similar both before (b) and after (c) the intercalation process, except for the absence of π-π* peak after the intercalation process. The presence of oxygen (d) was detected before and after the intercalation process, though the percentage is smaller after the intercalation. Fully resolved chlorine (e), and potassium (f) peaks in the intercalated sample allowed us to estimate the atomic percentage of potassium and chlorine as 4.77 and 0.7, respectively.

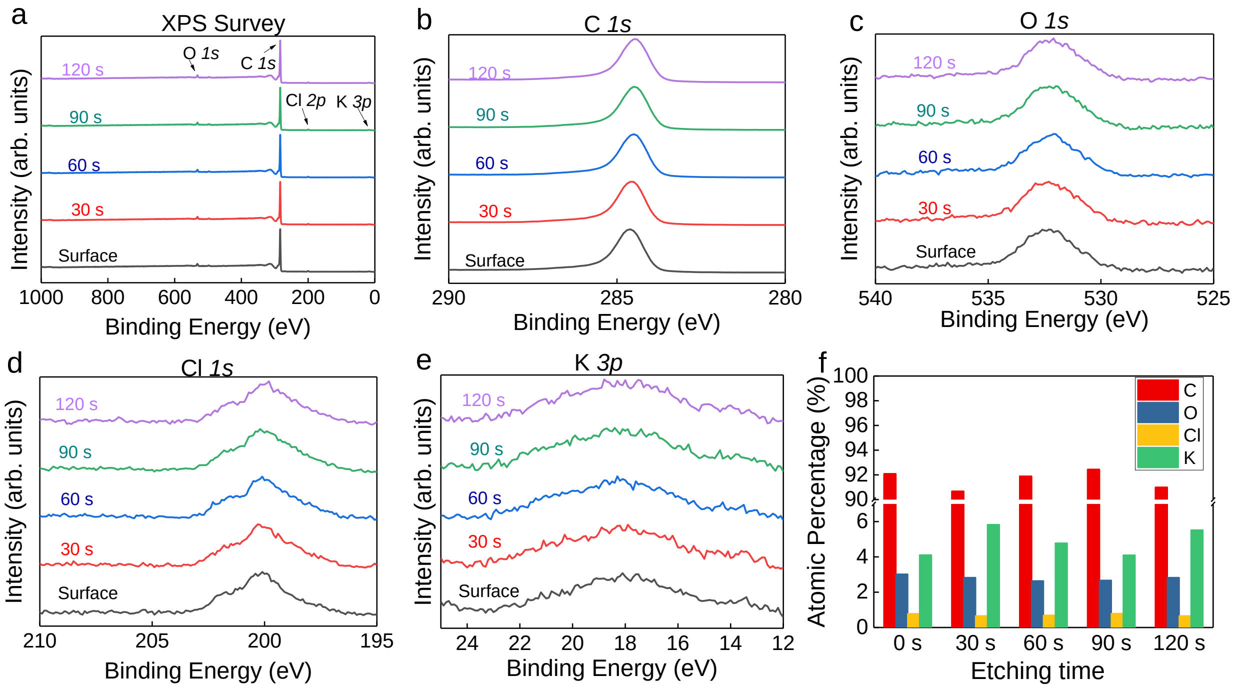

Supplementary Figure 8: XPS depth analysis. XPS data was collected at different depths of the intercalated graphite samples. XPS survey (a) and depth profiling of several elements found in ICG (b-e). (f) The atomic percentage of various elements present in ICG at different levels of depth.

Supplementary Note 7. Intercalation with water:

We have carried out a few other tests to see the effect of intercalant DI water on the expansion of the interlayer spacing of graphite and to see if the epoxy interface is stable after applying a high voltage for a long time. For this purpose, we used water as the intercalant instead of KCl and applied a voltage of 10 V for three hours. We utilized this sample for ionic conductance measurements at different concentrations of NaCl. In contrary to the samples intercalated with KCl, the water-intercalated samples exhibited very little enhancement in the current (Supplementary Figure 9). This experiment strongly suggests the importance of the large concentration of ions for successful intercalation. The negligible increase in current after the water intercalation also confirms the stability of epoxy glue. It indicates that even after applying a high voltage, there is no significant leakage in the samples.

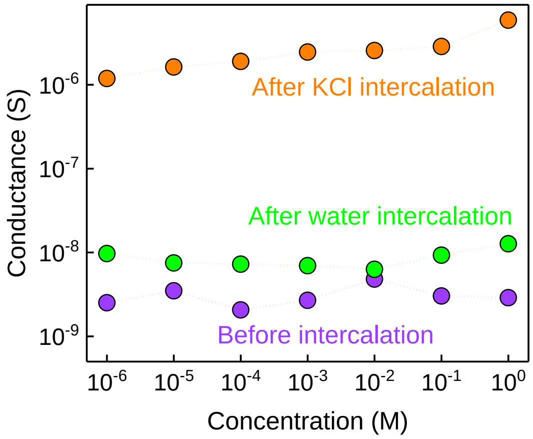

Supplementary Figure 9: Graphite intercalation with water and KCl. Comparison of conductance of graphite before and after the intercalation with water only and KCl only at several concentrations of NaCl. There is a clear enhancement in the ionic conductance through KCl intercalated samples, suggesting the importance of KCl in expanding the interlayer space.

Supplementary References:

1. Siria, A. *et al.* Giant osmotic energy conversion measured in a single transmembrane boron nitride nanotube. *Nature* **494**, 455–458 (2013).
2. Inagaki, M., Iwashita, N. & Kouno, E. Potential change with intercalation of sulfuric acid into graphite by chemical oxidation. *Carbon* **28**, 49–55 (1990).
3. Sheng-tao, Z., Anyan, G., Huan-fang, G. & Xiangqian, C. Characterization of Exfoliated Graphite Prepared with the Method of Secondary Intervening. *Int. J. Ind. Chem.* **2**, 123–130 (2011).
4. Livesey, R. G. *Foundations of vacuum science and technology*. (Wiley, 1998).
5. Cussler, E. L. & Cussler, E. L. *Diffusion: Mass Transfer in Fluid Systems*. (Cambridge University Press, 2009).
6. O'Hern, S. C. *et al.* Nanofiltration across Defect-Sealed Nanoporous Monolayer Graphene. *Nano Lett.* **15**, 3254–3260 (2015).
